# Identification and characterization of synthetic chondroitin-4-sulfate binding peptides in neuronal functions

**DOI:** 10.1038/s41598-018-37685-2

**Published:** 2019-01-31

**Authors:** Gabriele Loers, Yonghong Liao, Chengliang Hu, Weikang Xue, Huifan Shen, Weijiang Zhao, Melitta Schachner

**Affiliations:** 1 Center for Neuroscience, Shantou University Medical College, 22 Xin Ling Road, Shantou, Guangdong 515041 People’s Republic of China; 2 Center for Molecular Neurobiology, University Medical Center Hamburg-Eppendorf, Martinistraße 52, Hamburg, 20246 Germany; 3 Keck Center for Collaborative Neuroscience and Department of Cell Biology and Neuroscience, Rutgers University, 604 Allison Road, Piscataway, NJ 08854 USA

## Abstract

Chondroitin sulfate proteoglycans (CSPGs), up-regulated in and around the glial scar after mammalian spinal cord injury, have been suggested to be key inhibitory molecules for functional recovery by impeding axonal regrowth/sprouting and synaptic rearrangements. CSPG-mediated inhibition is mainly associated with the glycosaminoglycan chains of CSPGs, and chondroitin-4-sulfate (C4S) is the predominant sulfated structure that regulates axonal guidance and growth in the adult nervous system. With the aim to find molecules that neutralize the inhibitory functions of C4S, we screened a phage display library for peptides binding to C4S. From the phage clones binding to C4S we selected three peptides for further analysis. We observed that these peptides bind to C4S, but not chondroitin-6-sulfate, heparin sulfate or dermatan sulfate, in a concentration-dependent and saturable manner, whereas the scrambled peptides showed highly reduced or no binding to C4S. The C4S-binding peptides, but not their scrambled counterparts, when added to cultures of mouse cerebellar neurons and human neuroblastoma cells, neutralized the inhibitory functions of the C4S- and CSPG-coated substrate on cell adhesion, neuronal migration and neurite outgrowth. These results indicate that the C4S-binding peptides neutralize several inhibitory functions of CSPGs, suggesting that they may be beneficial in repairing mammalian nervous system injuries.

## Introduction

Mammals exhibit poor recovery after injury to the spinal cord due to the presence of growth inhibitors and diminished intrinsic regenerative capacity of mature neurons in the adult central nervous system^1–3^. The glial scar at and around the damaged area is generated by activated astrocytes and becomes a molecular and physical barrier impeding axonal regeneration^4,5^. A variety of cells, such as astrocytes, fibroblasts, microglia and oligodendrocyte precursor cells which are recruited to the injury site, participate in the formation of this glial scar. Interactions between inhibitors in the glial scar and neurons severely hinder axonal regrowth^6,7^. It is well accepted that glia-derived chondroitin sulfate proteoglycans (CSPGs) are major components of the extracellular matrix within the inhibitory glial scar^8^ and that inhibition is mainly associated with CSPG’s glycosaminoglycan chains. Much attention has thus been given to therapies aimed at removing the inhibitory properties of CSPGs, thereby providing improved functional recovery following spinal cord injury^9,10^.

CSPGs comprise a structurally diverse group of proteoglycans, consisting of a protein core to which glycosaminoglycans are covalently coupled. Chondroitin sulfate (CS) represents the predominant inhibitory glycosaminoglycan (GAG) structure that is expressed at and around central nervous system injury sites. CS consists of repeating disaccharide units composed of D-glucuronic acid (GlcA) and N-acetylgalactosamine (GalNAc), and can be modified by four different sulfotransferases that lead to synthesis of the following GAGs: CS-A, CS-C, CS-D, and CS-E. CS can be sulfated on carbon (C) 4 of GalNAc (CS-A), C6 of GalNAc (CS-C), C6 of GalNAc and C2 of GlcUA (CS-D), or C4 and C6 of GalNAc (CS-E)^11^. CS-A, which contains a high amount of C4S, is the predominant sulfation pattern in adulthood^12^ and negatively regulates axonal guidance and growth^13^. In the developing central nervous system, several different CSPGs appear to provide chemorepulsive signals to guide axonal growth^14,15^. After spinal cord injury, increased levels of CSPGs not only prevent the formation of new synaptic interactions, but also inhibit neuronal plasticity by blocking interactions between CS chains and the corresponding binding molecules^16^, thereby restricting action potentials and remyelination.

Among the methods that have shown promise in identifying ligands for functionally important molecules is the phage display technology, first introduced by George Smith^17^. This method represents a powerful and unbiased approach to identify peptide ligands for almost any target. Phage display is effective in producing up to 10^10^ diverse peptides or protein fragments^18–20^. The most frequently used system to date is the presentation of the peptides on the pIII protein of bacteriophage M13. Screening of phage display libraries benefits the most varied fields of research, such as peptide drug discovery^21^, isolation of high-affinity antibodies^22^, identification of biomarkers^23^, and vaccine development^24^.

In view of the expectation to find novel ways for identifying molecules that promote functional regeneration after injury, we aimed at identifying by phage display such molecules that neutralize the deleterious activities of C4S which is upregulated in expression after injury of the spinal cord; thirty seven peptides were identified showing high affinity to this glycan. We studied the effect of three of these peptides on neuronal cell adhesion and migration, and neuritogenesis through a series of *in vitro* experiments designed to analyze whether the C4S-binding peptides antagonize C4S inhibition, thereby providing a basis for a peptide-based therapy to ameliorate the devastating consequences of central nervous system injury.

## Results

### Identification of C4S-binding phages and determination of binding between identified peptides, C4S and CSPGs

**Figure 1:**
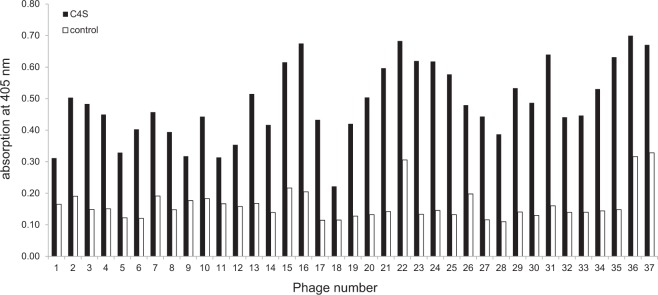
Identification of phages binding to C4S. To identify phages binding to C4S a phage display library was screened using immobilized C4S (150 μg/well) as substrate. After blocking with 1% BSA in PBS wells were incubated with 4 × 10^10^ phages in PBS, washed 10 times with 300 μl of TBS containing 0.1%, 0.3%, and 0.5% (v/v) Tween-20 in the first, second, and third rounds of panning, respectively. In each round of panning, binding phages were eluted with 100 μl of C4S (100 mg/ml in PBS) and applied to the next panning round. From 300 phages eluted after 3 panning rounds 37 were binding to C4S with high affinity.

To identify C4S-binding peptides a phage display library containing 10^9^ different filamentous phages presenting 12-mer peptides on the coat protein pIII was screened. Phages binding to immobilized C4S were eluted in three panning rounds using an excess of free C4S. The eluted 300 phage clones were subjected to a further ELISA and 37 clones showing the highest binding to C4S (Fig. 1) were picked and sequenced to determine the sequence of the peptides that they are carrying on their coat protein and that mediate the binding to C4S. Twenty-four positive phage clones were successfully sequenced. Eleven different peptide sequences were identified within the 24 phage clones and the peptide sequences were found 1 to 9 times (Table 1).

**Table 1:** Peptides encoded by phages binding to C4S. Sequences of the 12-mer peptides encoded by 24 phage clones showing highest binding to C4S are shown.

Frequency of occurrence	Peptide sequence	Name
3	AMDIAYRTHREP	AM
1	APNAPTQTTTPV	AP
1	ARHPDTNYSYGA	AR
2	DLNKPKPLYQQH	DL
2	ESMTYLSTAPEK	ES
9	GWVSNTTQAHHV	GW
2	HNYPVLRPNQIT	HN
1	MMKQTDQLLRNN	MM
1	NWHVYSTISNQT	NW
1	TRTPPESYASVR	TR
1	YSGKDLPPMKDS	YS

**Figure 2:**
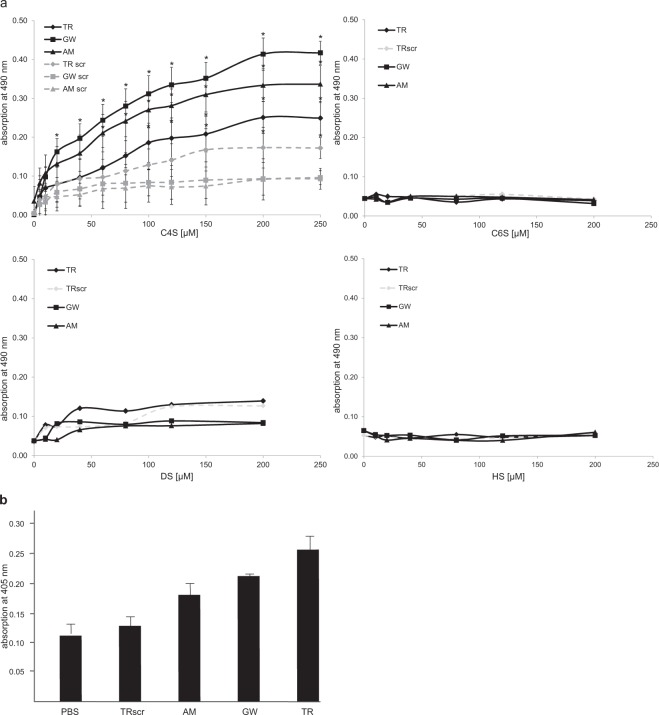
Concentration dependent binding of peptides to C4S and binding of TR, GW and AM to CSPGs. (**a**) Binding of biotinylated C4S-binding peptides AM, GW and TR and of their scrambled versions AMscr, GWscr and TRscr (5–200 µg/ml) to immobilized C4S, C6S, DS and HS (all at 100 µg/ml) was determined by detection of bound peptides with streptavidin-HRP. Experiments were done in triplicates and experiments were performed independently four to five times on C4S substrate and three times on C6S, HS and DS substrates. Mean values with SEM are shown (n = 4 for AM, GW, AMscr, GWscr and n = 5 for TR and TRscr on C4S and n = 3 for all peptides on C6S, HS and DS); One-way analysis of variance (ANOVA) with Tukey’s post-hoc test *p < 0.05 compared to scrambled controls. (**b**) Binding of biotinylated C4S-binding peptides TR, AM and GW and the scrambled peptide TRscr (200 µg/ml) to immobilized CSPG (100 µg/ml) was determined by detection of bound peptides with streptavidin-HRP. Experiment was done in triplicates and performed independently three times. Mean values with SEM are shown.

To verify binding of the peptides to C4S, the peptides occurring with the highest frequency (GW and AM) and the TR peptide showing the strongest binding in the phage ELISA were chosen and synthesized as well as their scrambled counterparts (TRscr, GWscr and AMscr) and a biotin label was added at the N-terminus of the peptides for detection. C4S was immobilized and binding of biotinylated peptides was determined using streptavidin-HRP. Results showed that the C4S-binding peptides GW and AM bound to C4S in a concentration-dependent and saturable manner, whereas their scrambled counterparts did not bind to C4S (Fig. 2a). In contrast, the C4S-binding peptide TR and its scrambled version showed concentration-dependent binding to C4S and the TR peptide bound only slightly better than the TRscr peptide (Fig. 2a). To confirm that the peptides specifically bind to C4S but not C6S, dermatan sulfate (DS) or heparin sulfate (HS), binding of the TR, GW and AM peptides to these glycans was analyzed. Results show that none of the peptides bound to C6S, DS or HS in a concentration dependent and saturable manner (Fig. 2a). As proof of principle, binding of the AM, GW and TR peptides and the scrambled TR peptide to CSPGs was determined and results showed that the TR, AM and GW peptides also bind to CSPGs as expected (Fig. 2b). Therefore, all peptides were considered to specifically bind to C4S and were chosen for further analysis and synthesized without a label.

### The C4S-binding peptides enhance neurite outgrowth on inhibitory CSPGs and C4S

**Figure 3:**
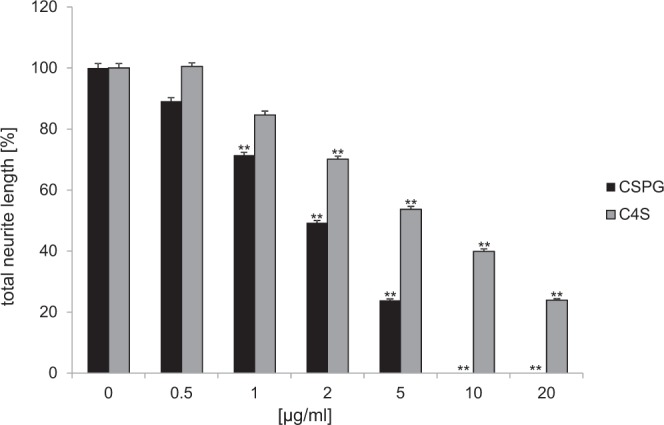
C4S and CSPGs inhibit neurite outgrowth of cerebellar granule neurons. Cerebellar granule neurons were seeded onto wells coated with PLL and with and without different concentrations of CSPGs and C4S (0.5–20 µg/ml) and neurite length were determined 48 h after seeding of cells. Histogram shows average values + SEM from four independent experiments (n = 4), **p < 0.01 (Student’s t-test).

**Figure 4:**
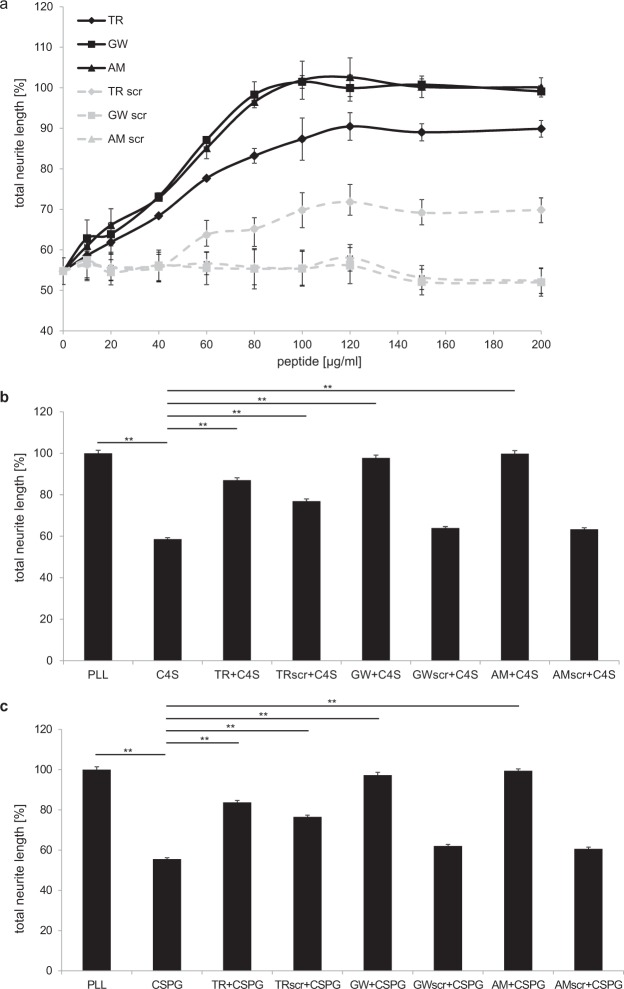
The C4S-binding peptides enhance neurite outgrowth in the presence of CSPGs and C4S. Cerebellar granule neurons were seeded onto wells coated with PLL followed by immobilization of 5 µg/ml C4S (**a**,**b**) or 2 µg/ml CSPG (**c**) and 30 min after seeding treated with PBS or peptides at 10–200 µg/ml (**a**) or 100 µg/ml (**b**,**c**) and neurite length were determined 48 h after seeding of cells. Histogram shows average values + SEM from four independent experiments (n = 4), *p < 0.05, **p < 0.01 (Student’s t-test).

CSPGs and C4S were shown to inhibit neurite outgrowth^13,25–28^. To evaluate the effects of the C4S-binding peptides on C4S and CSPG-mediated inhibition of neurite outgrowth, we measured the neurite length of cerebellar granule cells grown on C4S and CSPG substrate at 0.5–20 µg/ml. A concentration-dependent reduction of neurite outgrowth was detectable on CSPGs and C4S substrate, and was significant at 1, 2 and 5 µg/ml CSPGs and 2, 5, 10 and 20 µg/ml C4S (Fig. 3). At concentrations of CSPGs higher than 5 µg/ml neurite outgrowth of cerebellar granule neurons was totally inhibited. To study whether the C4S-binding peptides TR, GW and AM would block the neurite outgrowth inhibition C4S, cells were grown on poly-L-lysine (PLL) control substrate or 5 µg/ml C4S substrate, which reduced neurite outgrowth by approximately 50%, and were treated with PBS or C4S-binding peptides and scrambled peptides at 10–200 µg/ml (Fig. 4a,b). Neurite length was decreased in the C4S-treated group compared to neurite length on the control substrate PLL. Compared with neurons grown on C4S alone, the neurite lengths were increased when neurons were grown on C4S in the presence of the C4S-binding peptides at 40–200 µg/ml concentration. Highest effects were seen at 80–100 µg/ml concentration and did not increase further at higher peptide concentrations. Neurite outgrowth was totally rescued in the presence of the C4S-binding peptides AM and GW at 100 µg/ml and peptide TR was slightly less effective. Higher concentrations than 100 µg/ml of the TR peptide did not show a further increase in neurite outgrowth (Fig. 4a) and effects of the AM and GW peptides were still maximal. Interestingly, also the scrambled TR peptide reduced the growth inhibitory effect of C4S, although to a lesser extent than the TR peptide. Notably, scrambled sequences sometimes display an unplanned increase in functional efficiency. The scrambled peptides of GW and AM did not rescue neurite outgrowth on CSPGs and C4S substrates (Fig. 4a,b). Furthermore, the peptides GW, AM and TR (all at 100 µg/ml) as well as the scrambled peptide TRscr also increased neurite outgrowth on the CSPG substrate, whereas the scrambled peptides AMscr and GWscr did not have a significant effect (Fig. 4c). These results indicate that the C4S-binding peptides GW, AM and TR reduce the inhibitory effect of CSPGs and C4S on neurite outgrowth.

### The C4S-binding peptides neutralize the inhibitory effect of CSPGs on migration of SK-N-SH cells and of C4S on migration of cerebellar neurons

**Figure 5:**
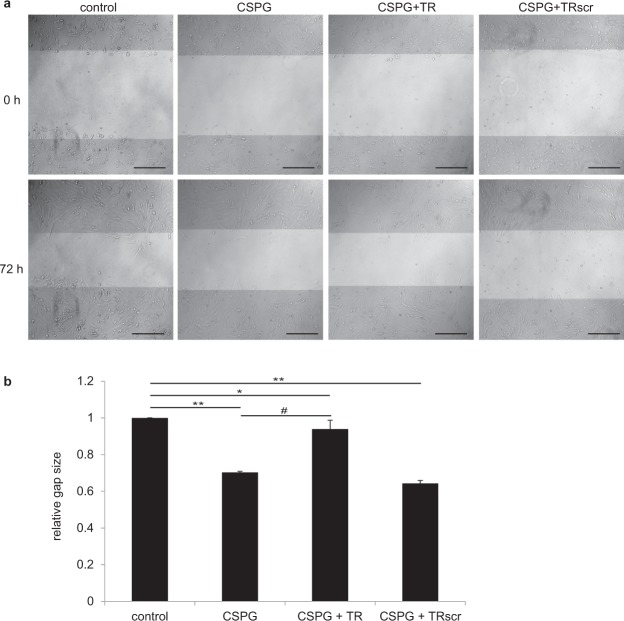
The C4S-binding peptide TR abrogates the inhibitory effect of CSPGs on SK-N-SH cell migration. SK-N-SH cells were grown for 12 h on CSPG substrate (2 µg/ml) or on CSPGs and TR or TRscr peptides (100 µg/ml) to form a confluent monolayer and then injured by scratching. Immediately after scratching (0 h) and after 72 h the size of the cell-free cleft and migration of cells into the cell-free scratched area were determined. Representative images show the cell-free area immediately after scratching (0 h) and 72 h after injury (**a**). Scale bar: 200 µm. (**b**) Histogram shows average values + SEM from six independent experiments (n = 6). **p < 0.01 for the CSPGs-treated group, *p < 0.05 for the TR- and TRscr-treated groups, respectively, versus vehicle control; ^#^p < 0.05 for the CSPG + TR-treated group, versus the CSPG-treated group (Student’s t-test).

Cell migration is an important process in the repair of the injured spinal cord, and CSPGs and CS-A which contains high levels of C4S have been shown to inhibit cell migration^25,29,30^. We therefore studied the effects of CSPGs and the C4S-binding peptide TR and its scrambled version TRscr on SK-N-SH cell migration in an *in vitro* wound assay. CSPGs (2 µg/ml) and the peptides (100 µg/ml) were immobilized and SK-N-SH cells were grown on top until confluency, injured by scratching and grown for further 72 h. Migration of SK-N-SH cells on CSPGs substrate and on CSPGs together with the scrambled peptide TRscr was inhibited (Fig. 5a,b). In contrast, migration of cells grown on CSPGs and the C4S-binding peptide TR was increased (Fig. 5a,b).

**Figure 6:**
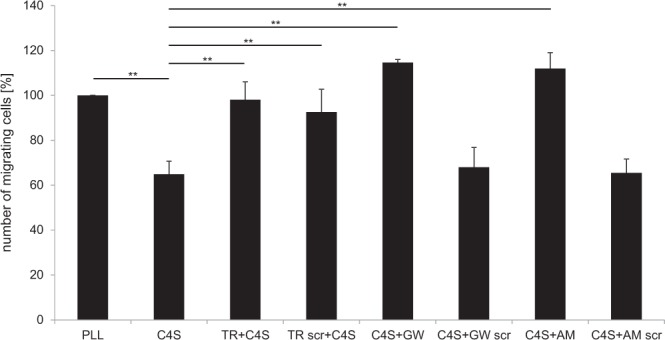
The C4S-binding peptides abrogate the inhibitory effect of C4S on cerebellar granule cell migration. Cerebellar explants were seeded onto wells coated with PLL or PLL followed by immobilization of 5 µg/ml C4S and treated with and without 100 µg/ml C4S-binding peptides and scrambled peptides and migration of cells was determined 48 h seeding of explants. Histogram shows average values + SEM from four independent experiments (n = 4), **p < 0.01 (One-way analysis of variance (ANOVA) with Tukey’s post-hoc test).

Migration of cerebellar granule neurons (CGNs) on C4S substrate (5 µg/ml) was reduced by 35.13% and normalized in the presence of the C4S-binding peptides TR, GW and AM at 100 µg/ml. Furthermore, the scrambled TR peptide also enhanced migration of CGNs on C4S, whereas the scrambled versions of peptides GW and AM showed no significant effect on neuronal migration on C4S. These results demonstrates that the C4S-binding peptides GW, AM and TR attenuate the inhibitory effect of CSPGs and C4S on cell migration (Fig. 6).

### C4S-mediated inhibition of neuronal adhesion and of migration of cerebellar granule cells is ablated in the presence of the C4S

**Figure 7:**
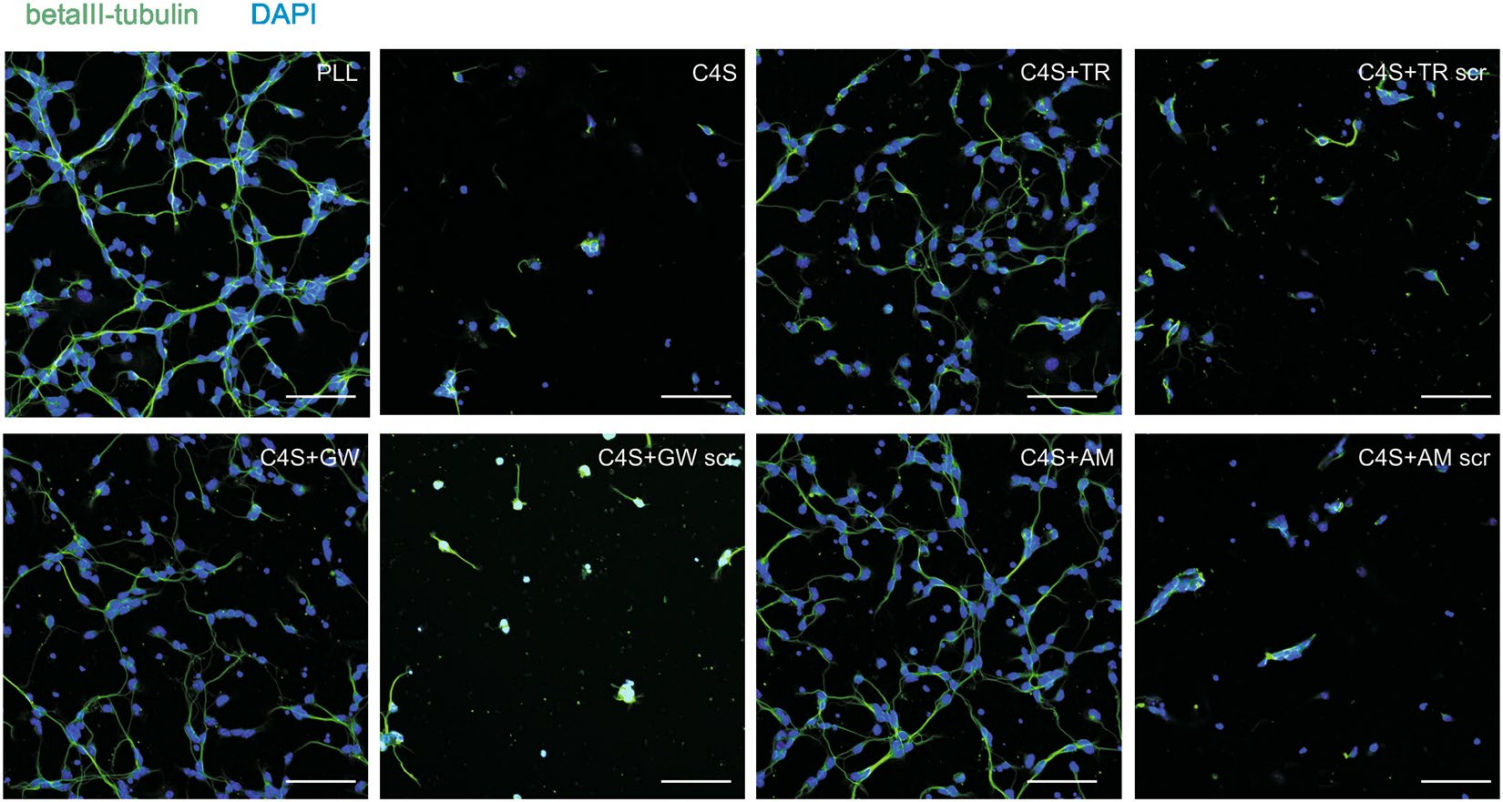
The C4S-binding peptides abolish the growth-inhibitory repellent effect of C4S. Cerebellar granule neurons were seeded onto PLL or C4S (10 µg/ml) and treated with C4S-binding peptides or scrambled peptides (100 µg/ml). After 48 h cells were fixed and stained against βIII-tubulin. Nuclei were visualized using DAPI. Representative images show cells with processes on C4S-substrate in the presence of the C4S-binding peptides but not in the presence of the scrambled peptides or in the absence of peptides. Scale bars: 20 µm.

Since CSPGs are a stop sign to a regenerating neuron, thereby displaying a non-permissive environment for growing axons^31^, we examined adhesion and growth of cerebellar granule cells on C4S substrate in the absence and presence of the C4S-binding peptides and scrambled peptides and on PLL in the presence and absence of peptides. The results showed that only few neurons were present on the C4S substrate and that these neurons displayed only very short neurites, when compared to neurons adhering and growing on PLL (Fig. 7). In the presence of the C4S-binding peptides, but not the scrambled peptides, the inhibitory effect of C4S on adhesion and outgrowth was abolished (Fig. 7). Furthermore, when neurons were maintained on PLL and treated with the peptides, their adhesion and outgrowth was not altered (data not shown). This result indicates that treatment with the C4S-binding peptides allows axons to extend on a growth inhibitory area of C4S and CSPG.

## Discussion

After spinal cord injury, the spared axons are capable of regrowing/sprouting. However, the glial scar at and around the lesion site is not only a mechanical barrier to regrowing axons, but contains inhibitory molecules that can convert regrowing axonal growth cones into a dystrophic ending that fails to extend within and beyond the lesion site. CSPGs are considered as major components of inhibitory molecules that restrict neurite growth and neuritogenesis. To neutralize the inhibition by molecules that ‘cover up’ and thereby silence the inhibitory sites, we screened a phage display library for peptides binding to C4S and identified thirty seven peptides with different sequences. From these peptides, we selected the most abundant and strongest binding peptides, and show that these peptides bind to C4S and CSPGs containing C4S but not to C6S, heparin sulfate or dermatan sulfate. Cell culture experiments showed that these peptides facilitate cell adhesion, neurite outgrowth and neuronal migration, indicating that they neutralize the inhibitory functions of C4S. Interestingly, the peptides do not affect neuronal cell adhesion, neurite outgrowth and neural migration on C4S always to the same extent. The scrambled version of peptide TR (TRscr) shows binding to C4S, although to a lesser extent than the TR, GW and AM peptides, and a positive effect on CGN migration and outgrowth on C4S substrate, but has barely an effect on CGN adhesion or on migration on SK-N-SH cells on C4S. These results suggest that not all cellular processes are mediated by the same C4S receptors or signaling pathways and that the peptide TRscr is only able to revoke some but not all of the pathways triggered by C4S. In addition, this peptide binds less efficiently to C4S and presents a scrambled order of amino acids found in peptide TR, so it had to be expected that this peptide is less efficient in reversing the inhibitory effects of C4S or that it should have no effect at all. Furthermore, the TR peptide showed stronger binding to CSPGs than the peptides AM and GW, suggesting that this peptide does not solely bind to C4S but might also bind to other CS structures present in the CSPGs.

CSPGs exert a variety of functions through the core protein, the number and location of CS-GAG chains, and through the extent of sulfation on GAGs. Much of the inhibitory effect of CSPGs is due to the activity of CS-GAGs^32^, with CSPGs having been identified as functional ligand for Nogo receptor 1^33^. The inhibitory properties of CS-GAGs that influence cell behavior have been determined^34,35^ in that chondroitinase ABC promotes functional regeneration of the injured spinal cord through degradation of CSs^36,37^. Since bacterial chondroitinase is a large molecule, the efficiency of its activity is confined by reduced diffusion in live tissue. Furthermore, this enzyme leads to immune reactions in the injected host. First attempts were taken to deliver a mammalian-compatible chondroitinase ABC and show a sustained and widespread digestion of CSPGs, resulting in significant functional repair as well as improved axon potential conduction and increased serotonergic innervation after spinal cord contusion injury in adult rats^38,39^. Nevertheless, it is important to develop small molecules that would be more efficient and easily applied. In precedence to our present study, C6S-binding peptides had been identified through peptide array screening and described to neutralize the C6S-mediated inhibition of cortical neurite outgrowth^40,41^. CSPG-mediated inhibition also occurs by interactions with receptors including protein tyrosine phosphatase σ (PTPσ), leukocyte common antigen-related (LAR) phosphatase, and Nogo receptors 1 and 3^42–45^. Another receptor for CSPG core proteins^46^ or CS-GAGs^47^ is the neural cell adhesion molecule (NCAM) which is involved in cell migration and survival, neurite outgrowth, and axon guidance^48–50^.

A peptide mimetic of the PTPσ wedge domain that binds to PTPσ, one of the identified receptors for the inhibitory glycosylated side chains of CSPGs, was shown to restore serotonergic innervation to the spinal cord below the level of injury and to facilitate functional recovery of both locomotor and urinary systems in the rat^51^. A variety of CSPG receptors have been suggested to provide new therapeutic targets aiming at prevention of CSPG-mediated inhibition after injury^11,43^, thus modifying CSPGs in combination with other therapeutic strategies is expected to be promising in promotion of spinal cord repair^11^. Indeed, regeneration after spinal cord injury can be promoted by degradation of CS on CSPGs^52^. Furthermore, genetic disruption of CS biosynthesis by targeting chondroitin polymerizing factor (ChPF) also increases axonal regrowth/sprouting^53^. The downstream targets of these CSPG receptors induce neurite retraction and growth cone collapse^54^ and impair neural stem cell migration through ROCK activation^25^. Activation of the Rho/ROCK pathway leads to phosphorylation of LIM domain kinase 2, myosin light chain, and other downstream proteins that induce cytoskeletal rearrangements such as neurite retraction and growth cone collapse^54,55^.

In accordance with previous studies showing that CSPGs and C4S inhibit neurite outgrowth and cell adhesion^25,27,34,56^, we now showed that the three C4S-binding peptides, but not their scrambled peptides block the inhibition of CSPGs/C4S on neurite outgrowth and cell adhesion. With the knowledge that protein tyrosine phosphatase PTPσ is activated upon binding of CS and inhibits neurite outgrowth^51,57,58^ it will be important to investigate whether the binding peptides will show similar effects on cellular functions in converting growth cones from a dystrophic state by stabilizing them within CSPG-rich substrates^5^. In addition, neurons growing on CSPGs were shown to upregulate integrin receptors^59^ and it was suggested that activating integrins may overcome CSPG inhibition in culture^60^. Therefore, in further studies the effects of the peptides on integrin expression and function should be determined.

Having identified three C4S-binding peptides and showing that these peptides reduce the inhibitory functions of CSPGs and C4S, it will be important to show how these peptides affect synaptic plasticity, structure and function of perineuronal nets, recovery from nervous system injury *in vivo* and how they impinge on signal transduction and on cytoskeletal elements in neurons.

## Materials and Methods

### Animals

C57BL/6 mice were purchased from the Guangdong Medical Laboratory Animal Center (People’s Republic of China) or bred at the animal facility of the University Medical Center Hamburg-Eppendorf and maintained at 22 °C on a 12 h light/12 h dark cycle and provided with food and water *ad libitum*. Mice of either sex were used for experiments. All experiments were conducted in accordance with the Chinese, German and European Community laws on protection of experimental animals, and all procedures used were approved by The Animal Ethics Committee of Shantou University Medical College (SUMC2014-004) or the responsible committee of The State of Hamburg (Permission Number Org_679). Experiments were carried out and the manuscript was prepared following the ARRIVE guidelines for animal research^61^ and all efforts were made to reduce the number of animals and their suffering in this work.

### Screening for peptides binding to chondroitin-4-sufate

For the screening procedure, the Ph.D.^TM^−12 phage display peptide library (New England BioLabs, Ipswich, MA, USA) was used. This library contains 1 × 10^13^ pfu/mL phages, with a diversity of 1.28 × 10^9^ unique peptide sequences and about 70 copies of each sequence. The library was used to express linear random 12-mer peptides fused to the N-terminus of M13 coat protein PIII. Three rounds of panning were performed as described^62^. Briefly, all wells of a heparin-binding 96-well plate (Corning, NY, USA) were coated by overnight incubation with 150 μl of C4S (1 mg/ml; A600300, Sangon Biotech, Shanghai, China) diluted in phosphate-buffered saline, pH 7.4 (PBS) at 4 °C. After blocking with 200 μl of 1% bovine serum albumin (BSA) in PBS for 60 min at room temperature (RT), the wells were incubated with 4 × 10^10^ phages in PBS for 60 min at RT. The wells were then washed 10 times with 300 μl of Tris-buffered saline (TBS; pH 7.4) containing 0.1%, 0.3%, and 0.5% (v/v) Tween-20 in the first, second, and third rounds of panning, respectively. In each round of panning, phages were eluted using 100 μl of C4S (100 mg/ml in PBS). Eluted phages were amplified and 2 × 10^11^ phages were applied for panning in the second and third rounds. After three rounds of panning, individual phage clones were isolated and single-stranded phage DNA was purified and subjected to DNA sequencing to determine the peptide sequences expressed by the phages.

### Phage ELISA

To verify binding of phage clones to C4S, wells were coated with 150 μl of C4S (1 mg/ml) or 150 μl of 1% BSA in PBS by incubation overnight at 4 °C. After blocking with 200 μl of 1% BSA in PBS for 60 min at RT, the wells were incubated with 4 × 10^10^ purified phages in PBS for 2 h at 25 °C. Wells were washed 6 times with 100 μl of PBS containing 0.03% Tween 20 and incubated with 100 μl of horseradish peroxidase (HRP)-conjugated M13 antibodies (GE Healthcare, Shanghai, China) in blocking solution for 60 min at RT. Wells were then washed 6 times with PBS containing 0.03% Tween 20, and finally 100 μl of 2,2′-azinobis-(3-ethylbenzthiazoline-6-sulphonate) was added. Absorbance was determined at 410 nm in a microtiter plate reader (Tecan Infinite M1000; Tecan, MA; USA).

### Peptide synthesis and peptide ELISA

To identify the C4S-binding peptides, 200 μl polyethylene glycol/NaCl (20% PEG8000 (w/v), 2.5 M NaCl) were added to phages that had bound to C4S and incubated at 26 °C for 20 min, then centrifuged at 8,600 g for 10 min at 4 °C. The pellets were resuspended in 100 μl iodide buffer (10 mM Tris-HCl, pH 8.0, 1 mM EDTA, 4 M sodium iodide) and 250 μl 100% ethanol was added, then incubated at 26 °C for 20 min, followed by centrifugation at 8,600 g for 10 min at 4 °C to pellet the DNA. The DNA pellet was suspended in 30 μl double distilled water and sent to Sangon Biotech (Shanghai, China) for sequencing. Based on the binding between phage clones and C4S, and our preliminary experiments testing the peptides in reversing the inhibitory role of C4S on neurite outgrowth and cell migration (data not shown), biotinylated (at the N-terminus) and non-biotinylated peptides AM (H-AMDIAYRTHREP-OH), GW (H-GWVSNTTQAHHV-OH) and TR (H-TRTPPESYASVR-OH) peptides, as well as their biotinylated and non-biotinylated scrambled counterparts AMscr (H-RDYHPARMITEA-OH), GWscr (H-STVQGAHTWVN-OH) and TRscr (H-TPTRSYPEVRAS-OH) were chosen for synthesis. The peptides were synthesized by Hanhong Biochemical Co. Ltd. (Shanghai, China) or Schafer-N (Copenhagen, Denmark).

A 384-well plate with high-binding surface was coated overnight at 4 °C with 25 μl C4S, C6S, heparan sulfate (HS) or dermatan sulfate (DS) (100 µg/ml; Sigma-Aldrich, Deisenhofen, Germany). After washing three times with PBS, the plate was incubated with 2% BSA in PBS for 90 min at RT. The plate was washed three times with PBS, and 25 μl PBS or 25 µl of biotinylated peptides AM, AMscr, GW, GWscr, TR and TRscr at 5–200 µM each were added and incubated for 90 min at 37 °C. The plate was washed with PBS containing 0.01% Tween-20 for five times and incubated with 100 μl HRP-coupled Streptavidin (Dianova, Hamburg, Germany; 1:1,000) for 60 min at 37 °C. After washing with PBS containing 0.01% Tween-20 five times, the plate was incubated with 25 μl ortho-phenylenediamine dihydrochloride (0.5 mg/ml) in the dark for 5 min at RT. The enzyme reaction was stopped by addition of 25 µl 2.5 M sulfuric acid per well and the absorbance was read using an ELISA reader at 490 nm (µQuant, Tecan, Bad Friedrichshall, Germany). All analyses were carried out in triplicate and the experiment was repeated four times (AM, GW, AMscr, GWscr peptides) or five times (TR and TRscr peptides) on the C4S substrate and three times on the C6S, HS and DS substrates for all peptides.

### SK-N-SH cell culture

Human neuroblastoma SK-N-SH cells (China Center for Type Culture Collection, Shanghai, China) were cultured at 37 °C and 5% CO_2_ in Dulbecco’s modified Eagle’s medium supplemented with 50 U/ml of a penicillin/streptomycin mixture and 10% fetal bovine serum (Sijiqing Biotechnology Co., Hangzhou, China).

### Adhesion, neurite outgrowth and migration of cerebellar granule neurons

Primary cultures of dissociated CGNs and cerebellar explants were prepared from cerebellum of 7-day-old mice as described^63,64^. Dissociated CGNs were seeded onto PLL-coated, CSPGs (2 µg/ml; Merck, Darmstadt, Germany) or C4S-coated (5 µg/ml; Sigma-Adrich, Deisenhofen, Germany) 48-well tissue culture plates or PLL-coated or C4S-coated glass coverslips in 24-well tissue culture plates at a concentration of 5 × 10^4^ cells/well (neurite outgrowth assay) or 1 × 10^5^ cells/well (cell adhesion and growth assay) in the presence or absence of 100 µg/ml of the non-biotinylated peptides if not indicated otherwise. Cells were incubated at 37 °C in 5% CO_2_ atmosphere for 24 to 48 h. To examine cell adhesion and gross cell growth CGNs were fixed with 4% formaldehyde for 30 min at 22 °C, blocked with PBS containing 2% BSA and 1% Triton X100 for 1 h at 22 °C and then stained with anti-βIII-tubulin monoclonal antibody (Santa Cruz Biotechnology, CA, USA; 1:1,000), followed by incubation with donkey anti-mouse secondary antibody conjugated to Cy2 (1:200). Cells were mounted using anti-fade mounting solution containing DAPI. Fluorescence images of CGN samples were collected using a confocal microscope (Olympus F1000, Olympus, Tokyo, Japan). To determine neurite outgrowth cells were fixed, stained and neurite lengths were quantified as described previously^63^.

Explants of 100 µm size were seeded onto PLL-coated or C4S-coated plastic coverslips in 24-well tissue culture plates and grown for 16 h in medium supplemented with 20% horse serum and with or without 100 µg/ml of the non-biotinylated peptides. After 16 h the serum was withdrawn and explants were cultured for further 32 h in the presence and absence of the peptides. Explants were then fixed and stained and migration of cerebellar neurons was quantified as described previously^65^. All experiments were performed in duplicates and four independent experiments were done for each assay.

### SK-N-SH cell migration assay

In order to investigate SK-N-SH cell migration on CSPGs in the presence and absence of C4S-binding peptide TR and the TR scrambled peptide, a scratch injury was conducted^66,67^. Briefly, CSPGs (2 μg/ml) with or without peptides (100 μg/ml) were coated onto 24-well tissue culture plates overnight at 4 °C. SK-N-SH cells were seeded onto the plates at a density of 5 × 10^4^ cells/well. After 12 h incubation the confluent monolayers were wounded by scratching the surface as uniformly as possible with a 200 µl pipette tip (resulting in a cell free area of about 800 µM), and fresh medium was added directly after scratching to remove floating cells. This initial wounding (0 h) and the movement of the cells into the scratched area were photographically monitored after 72 h incubation. For quantitative estimation of the distance between the borderlines, ten different equidistant points in each image were measured in order to better estimate the average width of the wounded area. The migration rate was expressed as the proportion of the mean distance between both borderlines caused by scratching, to the distance which remained cell-free after re-growing. One image was taken per well, and two wells were used per condition in six independent experiments.

### Statistical analysis

Statistical analysis was performed using the SPSS 17.0 (SPSS, Chicago, IL, USA) or Prism 5.0 software (GraphPad). All numerical data are presented as means ± SEM. Student’s t-test or One-way analysis of variance (ANOVA) with Tukey’s *post-hoc* test was used for comparisons as indicated. Values of p < 0.05 were considered statistically significant.
